# Opening new vista for rapid detection of antimicrobial resistant genes via film array multiplex PCR

**DOI:** 10.12669/pjms.39.6.8635

**Published:** 2023

**Authors:** Humaira Zafar, Irfan Ali Mirza

**Affiliations:** 1 Prof. Dr. Humaira Zafar, Professor of Pathology, Microbiologist, Al Nafees Medical College & Hospital, Isra University Islamabad Campus, Pakistan; 2 Prof. Dr. Irfan Ali Mirza, Professor of Pathology, Consultant Microbiologist, National University of Medical Sciences, Rawalpindi, Pakistan

The upsurge of antimicrobial resistance (AMR) along with its disastrous outcome had led to rank this situation amongst a list of Global health challenges. In April 2023, a joint testimony was formulated on 2^nd^ surveillance for AMR. It was a united effort by World Health Organization (WHO) and European Centre for Disease Prevention and Control (ECDC). The report showed red alarm for extremely high resistant pattern to last-line antibiotics especially the carbapenems in several countries of European Region.^1^ One published report for the year 2023 concluded that same burden of AMR can prime to approximate 10 million deaths each year by 2050.^2^

The extremely limited treatment options are becoming the main reason for high mortality rate especially in critically ill patients. This worsening scenario has compelled the scientists and researches around the Globe to search for it’s alternative management options. Till the availability of new pharmacological preparations, early diagnosis and prompt management can be the way out for reducing high morbidity and mortality rates. Likewise, non-availability of new drugs, conventional laboratory techniques are also time consuming to rapidly identify AMR cases, resulting in management delay. Comparatively the current situation for emerging trend of AMR requires an accurate, rapid, cost effective and point of care diagnostic tool for exact confirmation.

In these circumstances, availability of multiplex PCR via filmarray testing, came up with a solution for rapid detection of microbial etiology along with AMR gene. The methodology is proving as a breakthrough in the field microbiology for improving relevant diagnostics. Food and Drug Authority (FDA) USA has approved the usage of BioFire Filmarray for rapid and accurate diagnosis.^2^ Though a very costly tests, but weighing balance the cost with patient’s outcomes in terms of morbidity/mortality rate, duration of hospital stays, cost of ICU management and added health care burden, has forced it’s good diagnostic utility.

This novel approach harbors the ability to simultaneously identify the responsible microbe along with its AMR gene. It works on the principle of multiplexing the microbes in array. Various syndromic panels of BioFire film array have the ability to identify approximately 20 to 26 microbes and 10 to 12 AMR genes. So far, the available syndromic panels include Meningitis/ encephalitis, respiratory panel, pneumonic panel, gastro intestinal tract (GIT), and blood culture identification (BCID) panels. Besides BCID panel rest all can be performed by using direct clinical specimen. However, for BCID positive blood culture will be the mandatory requirement for proceedings. Furthermore, complete automation helps in reducing burden on lab personnel.^3^

The biofire blood panel helps to identify 27 targets. The Gram Positive bacteria include, Staphylococcus aureus, Streptococcus pneumonia, Streptococcus pyogenes, Streptococcus agalactiae, Enterococcus species, Listeria monocytogenes. The Gram negative bacteria includes Acinetobacter baumannii, Enterobacteriaceae, Klebsiella pneumonia, Klebsiella oxytoca, Escherichia coli, Proteus species, Pseudomonas aeruginosa, Enterobacter cloacae complex, Neisseria meningitides, Haemophilus influenzae, Serratia marcescens. The yeast includes Candida albicans, Candida tropicalis, Candida glabrata, Candida krusei, Candida parapsilosis. The AMR genes include methicillin resistant genes (RG) i.e mecA mecC, mecA/C and mec right- extremity junction (MREJ), vancomycin resistant genes i.e van A/B, carbapenemases resistant genes i.e imipenem (IMP), RG for Klebsiella pneumoniae carbapenemase (KPC), oxacillinase (OXA-48) resistant genes, RG for New Delhi metallo-β-lactamase (NDM), Verona integron-encoded metallo-β-lactamase (VIM) resistant genes, RG for extended spectrum beta lactamase (ESBL) i.e cefotaxime (CTX-M) and resistant genes for colistin i.e mobilized colistin resistance (mcr-1) and.^4^

The BioFire respiratory panels helps to identify 21 targets. The viruses includes, Adenovirus, Coronavirus 229E, Coronavirus OC43, Coronavirus NL63, Coronavirus HKU1, Influenza A, Influenza B, Influenza A/H1, Influenza A/H1- 2009, Influenza A/H3, Parainfluenza viruses (PIV)- 1, PIV-2, PIV-3, PIV-4, Respiratory syncytial virus, Human Meta pneumovirus, Human Rhinovirus and Enterovirus. The bacteria includes Bordetella pertussis, Bordetella parapertussis, Mycoplasma pneumoniae and Chlamydia (Chlamydophila) pneumonia.^4^

The pneumonia panel helps to identify 33 targets including various bacteria, viruses and AMR genes. The semi-quantitative bacteria includes, Acinetobacter calcoaceticus baumannii complex, Klebsiella pneumoniae, Klebsiella oxytoca, Klebsiella aerogenes, Pseudomonas aeruginosa, Streptococcus pneumonia, Haemophilus influenzae, Moraxella catarrhalis, Proteus species, Enterobacter cloacae complex, Escherichia coli, Serratia marcescens, Staphylococcus aureus, Streptococcus pyogenes , and Streptococcus agalactiae. The atypical qualitative bacteria includes Chlamydia pneumoniae, Mycoplasma pneumoniae and Legionella pneumophila. List for viruses include Coronavirus, Adenovirus, Respiratory syncytial, Influenza A & B, Parainfluenza, Human Rhinovirus, Human Metapneumovirus, and Enteroviruses. The targeted AMR genes includes mec A/C, MREJ, IMP, OXA-48, KPC, VIM, NDM, ESBL and CTX-M.^4^

The meningitis/ encephalitis panel helps to identify 14 targets. The bacterial list include Neisseria meningitides, Streptococcus pneumonia, Haemophilus influenzae, Escherichia coli, Listeria monocytogenes and Streptococcus agalactiae. The viruses include Cytomegalovirus (CMV), Varicella zoster Virus (VZV), Herpes simplex Virus 1 (HSV-1), HSV-2, HSV-6, Human Parechovirus and Enterovirus. The yeast include Cryptococcus neoformans and Cryptococcus gattii.^4^

The GIT panel of BioFire Filmarray facilitate identification of 22 targets. The bacteria includes Clostridium difficile (toxins A & B), Campylobacter species (jejuni, coli, and upsaliensis), Salmonella species, Vibrio (parahaemolyticus, vulnificus, and cholerae), Plesiomonas Shigelloides, Yersinia enterocolitica. The Diarrheagenic Escherichia coli (E.coli) and Shigella species include *E.coli* O157, Enteroaggregative *E.coli* (EAEC), Enterotoxigenic *E.coli* (ETEC), Enteropathogenic *E.coli* (EPEC), Enteroinvasive *E.coli* (EIEC) and Shiga-like toxin-producing *E.coli* (STEC) stx1/stx2. The viruses includes Rotavirus A, Norovirus GI/GII, Adenovirus F40/41, Sapovirus (I, II, IV, and V) and Astroviruses. The parasites includes Entamoeba histolytica, Giardia lamblia, Cryptosporidium and Cyclospora cayetanensis.^4^

Contrary narrating by flashing back to the knowledge about conventional diagnostic techniques used in microbiology highlights firstly the gold standard culture and sensitivity testing. Though ideal for utilization, but came up with the problem of time-consuming proceedings. Furthermore, the proceedings are dependent upon many resources i.e. manpower, technicalities, machinery etc. Moving forward, comes up the serological diagnosis, molecular level identification, hybridization assays, and utility of nano diagnostic tools. Talking for every single tests comes up with pros and cons for their selection. Concluding all, whatever the main category, the results will mainly address the single microbe along with its susceptibility pattern. Thus, all the delay will worsen the sufferings of AMR patients.^2^

This all compels the strengthening of infection prevention and control (IPC) committees of hospitals. Their focus should be the strict of recommended IPC guidelines and adherence to anti- microbial stewardship programs. In view of the deteriorating circumstances due to AMR and its high mortality outcomes, establishment of National and International diagnostic and management plans are the dire need of time. The united efforts by the health care providers, health policy makers, researchers and Government should endorse implementation of all those plans. Allocation of desired extra funds by the Government to strengthen microbiological diagnostic facilities are the need of time to coup up with this Global challenge.^5^ The Film Array Multiplex PCR technology is a transformative contrivance in the battle against AMR and its devastating outcomes. Thus, facilitation for the incorporation of this diagnostic technique holds a promising role for the rapid and accurate diagnosis of AMR cases. The resultant of all these united efforts will be to combat AMR and reducing the sufferings of patients in that perilous scenario.

## Author’s Contribution:

**HZ:** Finalization of entire manuscript & Corresponding author. **AIM:** Topic selection, Supervising write up for entire manuscript and it’s finalization prior submission.
